# Supplementary material for: Studying regional low-carbon development: A case study of Sichuan Province in China
Source: PLoS One. 2024 May 29;19(5):e0302733. doi: 10.1371/journal.pone.0302733 (PMC11135781; doi:10.1371/journal.pone.0302733)
Supplement: Attachment — Submitted filename: Response to Reviewers-PONE-D-23-37224.docx [file pone.0302733.s002.docx]

**Response to Reviewers’ Comments**

We appreciate very much for your decision and constructive comments on our manuscript. These comments are very helpful for us to revise this paper. We have carefully considered the suggestions of the Reviewers, and tried our best to carry out a comprehensive revision to improve the manuscript. The revision notes are addressed point by point below.

**Response to Reviewer 1 Comments**

**Point 1:** State of art shall be mentioned more clearly

**Response 1:** Thank you for your careful review and valuable suggestion. We have revised the manuscript to provide a clearer and more comprehensive overview of the state of the art in the relevant field. Specifically, we have summarized the current mainstream viewpoints and research directions in the field, while also highlighting the existing shortcomings and unresolved issues. Additionally, we have explicitly stated the specific research questions and objectives that our study addresses, aiming to provide readers with a clearer understanding of the background and positioning of our research. The above-mentioned modifications are available in Lines 75-142 in Section 1.

**Point 2:** Practical utilization should be discussed further at the end of the discussion section

**Response 2**: Thank you very much for your suggestion. We have adjusted the section “5.2 Policy Implications” to “4.3 Policy Implications”, and added a dedicated paragraph that emphasizes the potential application prospects and the inherent value of our research outcomes (Lines 427-458). This includes discussions on how to address the unresolved issues that future research should focus on. Furthermore, we have highlighted how our research can contribute to the advancement of the field.

**Point 3:** Limitation of the research shall be acknowledged

**Response 3**: Thank you very much for your suggestion. In response to this comment, we have included the section “4.4 Scope for Future Research”, available in Lines 459-468 in the Manuscript. In the section, we explicitly acknowledged the limitations and shortcomings of our research. This includes discussing the limitations related to sample sizes and the potential shortcomings of the research methods employed, the simplifications in research assumptions, and the scope limitations. By addressing these limitations, we aim to provide a balanced and transparent presentation of our study.

**Point 4:** Update introduction get insight with the available resources and perspective of urban climates for example: https://doi.org/10.1016/j.scs.2023.104459 ; Urban Climate 51, 101637 ;Also Consider incorporating insights from sources https://doi.org/10.17660/ActaHortic.2023.1374.14 and https://doi.org/10.17660/ActaHortic.2023.1374.11 ; Land 12 (8), 1623 ;to acknowledge the various benefits of green spaces relevant to this study.

Response 4: Thank you for the valuable references. We have updated and enriched the introduction section by incorporating insights from the provided references, available in Lines 35, 37 in the Manuscript. Specifically, we have integrated perspectives from the referenced papers to enhance the discussion of the current research progress and viewpoints in the field of urban climates. Additionally, we have incorporated the various benefits of green spaces relevant to our study, such as temperature reduction and pollution absorption, based on the insights from the provided sources. We have also updated the reference section to include these additional sources, thereby enriching the background of our research.

**Point 5:** Improve the figures quality e.g., figure 5,7,…

**Response 5:** Thank you very much for your suggestion, we have made the following changes to address the issue of chart quality, firstly, to reduce the problem of image clarity degradation during file transfer, we have updated all charts to the Scalable Vector Graphics format. Secondly, to improve the aesthetics and clarity of the charts, we have adjusted the colour scheme of the charts.

**Point 6:** Number of references is low.

**Response 6:** Thank you for your suggestions. To make the paper more in-depth and informative, we have added other 12 citations. In total, there are 51 citations throughout the paper. By adding these citations, we have compared the content of this paper with previous research, thus increasing the scientific validity and credibility of our study.

**Point 7:** Re-organize the manuscript. I suggest 1. Introduction 2 methods 3 results 4 discussion (include practical utilization and limitations) 5 conclusion

**Response 7:** We are grateful for the suggestion. To make the paper more rigorous, it has now been structured as follows: Section 1 Introduction, Section 2 Methods, Section 3 Results, Section 4 Discussions, and Section 5 Conclusions.

**Response to Reviewer 2 Comments**

**Point 1:** Abstract section: Lines 16 to 19. it is difficult to understand the status in different periods via the expression. Please do not express such results in subordinate clauses.

**Response 1:** We appreciate the feedback regarding the clarity of expression in the abstract section. In response to this, we have revised the mentioned lines to improve the comprehensibility of the status in different periods. Specifically, we have extracted the content from the subordinate clause and presented it as standalone sentences. By directly stating the research status in different periods and avoiding complex grammatical structures, we aim to enhance the clarity and ease of understanding for readers.

**Point 2:** In the introduction section, the authors describe a lot of research background, especially the presentation of some data. However, I would have preferred to see the significance of the study, such as the authors' statement that it is the first study and fill a research gap.

**Response 2:** Thank you for your careful review and valuable suggestion. We have restructured and enriched the introduction section to address the significance of the study as suggested. Specifically, we have included a concise description of the necessary research background knowledge while emphasizing the originality and innovation of our study . Additionally, we have explicitly stated the significance and contributions of our research, including how it fills existing research gaps. We have clarified the research objectives and tasks, and we have utilized data to support our points effectively. Furthermore, we have highlighted the potential impact of our research on both academic and practical domains.The above-mentioned modifications are available in Lines 114-142 in Section 1.

**Point 3:** Line 88. what does OECD mean?

**Response 3:** Thank you for your careful review. OECD is the abbreviation of the Organization for Economic Co-operation and Development. While keeping the language simple and fluent, we have made the necessary changes to the abbreviation with its full name. In addition, we checked other abbreviations in the paper to ensure that they were accompanied by a clear explanation the first time they appeared.

**Point 4:** All the tables and figures, please add the units if it is needed.

**Response 4:** We think this is an excellent suggestion. We checked the images and tables in the paper and labelled the data that required the use of units with the appropriate units as a way of presenting our findings more accurately.

**Point 5:** Discussion section, I would like to see the theoretical implications of the paper. For instance, comparisons and enhancements with related studies.

**Response 5:** Thank you for your suggestions. In order to illustrate the practical and theoretical significance of this paper, we have added the related contents in the Discussion section (Lines 431-436). Specifically, in terms of theoretical significance, this study enriches the research content of regional economics by illustrating the intricate and dynamic relationship between economic growth, energy consumption and carbon emissions through a detailed case study of Sichuan Province. In addition, this study contributes to a deeper understanding of the mechanisms behind the optimisation of energy efficiency and industrial structure, and provides valuable insights for the formulation of sustainable development policies.

**Point 6:** This paper has yet to be polished.

**Response 6:** Thank you for your suggestions. After fully considering the valuable comments of the reviewers, we have revised the paper carefully, by checking the grammar, modifying appropriate words, optimizing sentence structures and so on.

**Point 7:** Line 272. It is strange that Fig.2 does not show any GDP information. I find that in Figure 3, but the most key point is, why the GDP curve has a great decrease in 2020? it is so strange. please also check the curve of carbon emission.

**Response 7:** Thank you for your careful review. We have checked the content of Figures 2 and 3 and found that the statement at Line 272 was a chart labelling error and we have corrected it accordingly (Line 307, the new Fig. 2 in Lines 72-74). Regarding your suggestion to check the GDP data, we are very concerned about it. After checking, we found that it was a data editing error in the process of making the charts, and we have corrected the GDP data in Fig. 2. In order to avoid the a error, we have rechecked and corrected all the data in the full text.

**Response to Reviewer 3 Comments**

**Point 1:** The manuscript needs major revision due to its uncategorized and insufficient Introduction and Method section. Introduction needs more clear notice regarding the topic.

**Response 1:** Thank you for your review and constructive comments on our manuscript. We appreciate the suggestions you made to strengthen key sections. In response to point 1 about the need for a clearer introduction to the topic, we have thoroughly revised the introduction section. We clearly stated the research objectives and questions. We also provided a detailed review of relevant theories and research and clearly articulated the innovations of the study.

We also revised the methodology section. To make the use of methods clearer and more explicit, we added empirical thinking (Lines 149-155). This section describes which elements we primarily use TAPIO and LMDI to demonstrate. In addition, we have adjusted the language of the methodology section so that our use of the methodology can be expressed more clearly.
