# Supplementary material for: Studying regional low-carbon development: A case study of Sichuan Province in China
Source: PLoS One. 2024 May 29;19(5):e0302733. doi: 10.1371/journal.pone.0302733 (PMC11135781; doi:10.1371/journal.pone.0302733)
Supplement: Attachment — Submitted filename: Response to Reviewers-PONE-D-23-37224-0407.docx [file pone.0302733.s003.docx]

**Response to Reviewer’s Comments**

We would like to thank you for your professional review work, constructive comments, and valuable suggestions on our manuscript. Your time and efforts are greatly appreciated. We have strived to improve the manuscript accordingly as listed in details below.

**Point 1:** The conclusion should be shorter, preferably one paragraph, concise, and depict the broader message of the study.

**Response 1:** Thanks to your careful review and valuable suggestions, we have streamlined the conclusion section of the article. Mainly, we have consolidated the expression of 469-495 lines in the previous draft into a one-paragraph expression of 469-489 lines, and the word count has been streamlined from 362 words to the current 283 words. Considering the content of the conclusion section needs to depict the broader message. We conclude with a brief summary of the paper’s findings, research contributions, research limitations, and suggestions for future research directions. Due to the large amount of information that needs to be presented, it has been streamlined as much as possible, leaving the content that is important and needs to be explained in the conclusion.

In addition, we have rechecked the manuscript again and improved some details, available in Revised the Manuscript with Tracked Changes-PONE-D-23-37224.
